# Defining multiplicity of vector uptake in transfected *Plasmodium* parasites

**DOI:** 10.1038/s41598-020-67791-z

**Published:** 2020-07-02

**Authors:** Manuela Carrasquilla, Sophie Adjalley, Theo Sanderson, Alejandro Marin-Menendez, Rachael Coyle, Ruddy Montandon, Julian C. Rayner, Alena Pance, Marcus C. S. Lee

**Affiliations:** 1 Wellcome Sanger Institute, Wellcome Genome Campus, Hinxton, UK; 2 Department of Immunology and Infectious Diseases, Harvard T.H. Chan School of Public Health, Boston, USA; 3 The Francis Crick Institute, London, UK; 4 MIVEGEC, IRD, CNRS, University of Montpellier, Montpellier, France; 5 Wellcome Centre for Human Genetics, Oxford, UK; 6 Cambridge Institute for Medical Research, University of Cambridge, Cambridge, UK

**Keywords:** Biological sciences, Microbiology, Parasitology, Parasite genomics

## Abstract

The recurrent emergence of drug resistance in *Plasmodium falciparum* increases the urgency to genetically validate drug resistance mechanisms and identify new targets. Reverse genetics have facilitated genome-scale knockout screens in *Plasmodium berghei* and *Toxoplasma gondii*, in which pooled transfections of multiple vectors were critical to increasing scale and throughput. These approaches have not yet been implemented in human malaria species such as *P. falciparum* and *P. knowlesi*, in part because the extent to which pooled transfections can be performed in these species remains to be evaluated. Here we use next-generation sequencing to quantitate uptake of a pool of 94 barcoded vectors. The distribution of vector acquisition allowed us to estimate the number of barcodes and DNA molecules taken up by the parasite population. Dilution cloning of *P. falciparum* transfectants showed that individual clones possess as many as seven episomal barcodes, revealing that an intake of multiple vectors is a frequent event despite the inefficient transfection efficiency. Transfection of three spectrally-distinct fluorescent reporters allowed us to evaluate different transfection methods and revealed that schizont-stage transfection limited the tendency for parasites to take up multiple vectors. In contrast to *P. falciparum*, we observed that the higher transfection efficiency of *P. knowlesi* resulted in near complete representation of the library. These findings have important implications for how reverse genetics can be scaled in culturable *Plasmodium* species.

## Introduction

Reverse genetics is a key tool in the global effort to identify drug targets or resistance mechanisms, as well as to explore new biology. Technologies for genetic manipulation of organisms have advanced significantly in the last decade, particularly through site-specific nucleases such as Cas9^1^ that can be used to increase the efficiency and specificity of modification. However, using genetics to validate gene function in *Plasmodium falciparum,* the most virulent of the causative agents of human malaria, has been consistently challenging, for multiple reasons. The high AT content of its genome (> 80%) makes generating large stable plasmids in *E. coli* difficult, and also limits the potential targets for Cas9 due to its requirement for an NGG protospacer adjacent motif (PAM) sequence, which is much rarer in *P. falciparum* genomic DNA than most eukaryotes. *P. falciparum* also has low transfection efficiencies compared with other *Plasmodium* species^2,3^, despite attempts to generate more efficient protocols^4,5^. These constraints have limited progress in interrogating the genome of the parasite to uncover potential new drug targets and the roles of the many genes of unknown or poorly described function.

There are also specific challenges to the application of CRISPR/Cas9 to large-scale genetic screening in *P. falciparum*, unlike in related apicomplexans such as *Toxoplasma*^6^. Analysis of the *P. falciparum* genome^7^ indicates that this organism lacks one of the two major DNA repair mechanisms, non-homologous end joining (NHEJ). In principle, this should provide an advantage for genome editing, as the introduction of a double strand break in the parasite DNA coupled with homology templates that provide the desired modification should result in consistent homology-directed repair without competing error-prone events. As a result, the application of CRISPR/Cas9 in *P. falciparum* has relied on donor templates and has been used to knockout genes^8,9^, and validate key drug resistance mechanisms^10–12^. However the absence of NHEJ, coupled with the low transfection efficiency noted above, has made systematic large-scale gRNA-based gene disruption screens elusive^6,13^. Newly developed CRISPR-interference and CRISPR-activation approaches^14–17^, which use a nuclease-dead version of Cas9 as a DNA-binding protein to either repress or activate gene expression, might allow multiplexed screening even in the absence of NHEJ machinery. However, such approaches will rely on achieving relatively high transfection efficiencies, and an understanding of how many unique plasmids (containing different gRNAs) are taken up by each parasite.

While *P. falciparum* co-transfection is routinely performed with two or three plasmids that each have unique selectable markers, to our knowledge the dynamics of *P. falciparum* transfection have not been studied with techniques that allow the multiplicity of vector uptake to be accurately quantitated. We sought to measure the propensity of *P. falciparum* to take up multiple plasmids by transfecting pools of vectors that could be distinguished either on the basis of sequence or on the fluorescent properties of their encoded products. We then examined both the proportion of these vectors taken up by parasites in bulk culture, and how these events were distributed within cloned parasite populations. In one set of experiments we co-transfected 94 vectors that each encoded a unique DNA barcode, and used next generation sequencing to quantitate their uptake in the bulk parasite population as well as in cloned transfected parasites. In a second approach, we generated a set of expression vectors encoding fluorescent reporter proteins and used these to evaluate different transfection methods—preloading of red blood cells, ring-stage transfection, or schizont-stage transfection—to detect biases in multiplicity of plasmid uptake. We also examined the transfection properties of a different *Plasmodium* species, *P. knowlesi*, which is reported to possess a considerably higher transfection efficiency than *P. falciparum*^3^, to determine if this system would yield a richer representation of the barcode pool. The data from these collective approaches help us to understand the limitations of the species and the transfection method applied, and have important implications for the design of future library-based genetic experiments.

## Results

### Transfection of a pooled barcode library into *P. falciparum*

**Figure 1:**
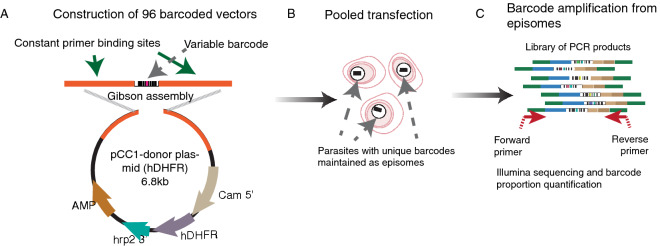
Graphical representation of the plasmid assembly method using an amplicon containing 96 unique barcodes amplified from a library of pJazz Knockout vectors^2^. (**A**) Amplicon assembly of all 96 barcodes into the pCC1-hDHFR transfection vector was performed in a single Gibson reaction, and the bacterial transformation was used to directly seed a large overnight culture for midiprep plasmid preparation. This starting pool was examined by Bar-Seq to confirm representation of barcodes (see Fig. 2). (**B**) Pools of 94 constructs were used as DNA input for transfection. (**C**) Parasite genomic DNA was extracted from both bulk cultures and individual clones for barcode amplification from episomes for Next Generation Sequencing and barcode quantification.

In order to examine the diversity of vector uptake after transfection, we created a large pool of plasmids that differed only in a short 11 bp barcode, allowing identification of each plasmid uniquely by next generation sequencing, but minimising differences in sequence that may bias their propagation in the parasite or subsequent amplification by PCR. Using a library of barcoded vectors would allow us to address the complexity of plasmid representation that could be achieved after transfection, and also the multiplicity of plasmid uptake in individual parasite clones. The pool of vectors was created by first amplifying 96 unique DNA barcodes within a short 120 bp cassette^18^ and assembling them, in a single Gibson reaction, into a vector backbone that contained the h*DHFR* selectable marker, allowing for positive selection (Fig. 1a). This vector pool was transfected into ring-stage parasites from two standard *P. falciparum* laboratory strains, 3D7 and Dd2, and constant drug pressure with WR99210 was applied the following day. Once parasites became visible by Giemsa stain, the cultures were expanded, and parasite genomic DNA was extracted. Barcodes were amplified by PCR and quantitated by Illumina sequencing (Fig. 1b,c) using the barcode sequencing method developed in *P. berghei*^19^. The starting vector pool was also sequenced to allow for comparison of barcode distributions before and after transfection. The transfection was repeated on five independent occasions.

**Figure 2:**
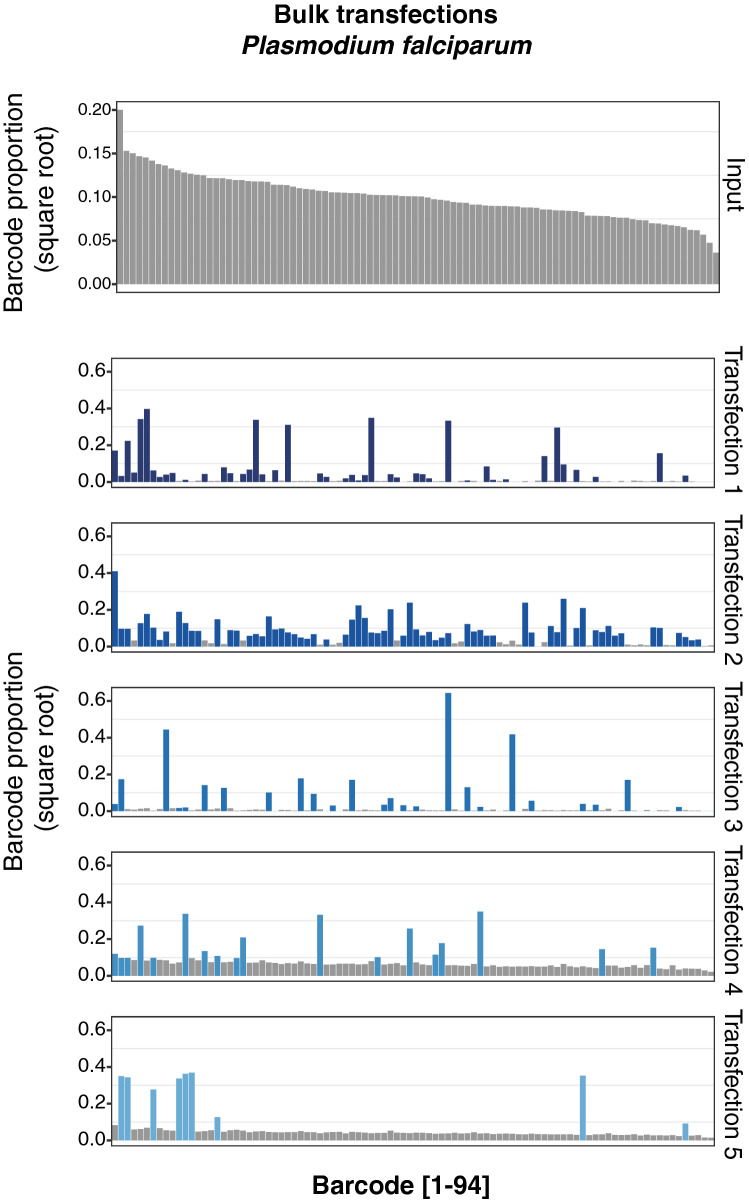
Transfection of a pool of 94 barcoded vectors into *P. falciparum*. Individual barcodes are represented on the x-axis, and the data is sorted by the most abundant barcodes present in the input pool (first panel, grey). The y-axis represents the square root of the proportion of each barcode in the total pool, per individual transfection as obtained by Next Generation Sequencing. For each transfection, bars colored in blue represent the most abundant barcodes, reflecting successful uptake as defined in Table 1.

Ninety four of the 96 barcodes were represented in the input pool used for transfection (Fig. 2, Input), albeit at differing frequencies. However, examination of the bulk population of transfected parasites indicated that not all barcodes had been taken up by parasites and maintained as episomes. To quantify the number of unique barcodes taken up, as well as to estimate the total number of plasmids acquired, for each barcode we calculated the difference between its log-ratio in the input and in the final population of parasites. This resulted in a bimodal distribution as shown (Supplementary Information), which we took to represent a superposition of two normal distributions: one representing barcodes successfully taken up by parasites and another representing barcodes that were not. In each case we fitted a mixture of these two distributions to estimate the number of unique barcodes in each transfection. By simulating random sampling of barcodes with the relative proportions found in the input, we were able to estimate the number of molecules taken up in each transfection, which ranged from 10 to 131 (Supplementary Information). This translated to a predicted range of 9–66 unique barcodes represented in the bulk populations (Table 1).

**Table 1:** Modelled values for the number of barcodes taken up in each *P. falciparum* transfection, and the expected number of molecules of DNA acquired that this corresponds to, given the possibility that one barcode could be taken up multiple times.

Transfection	Unique barcodes	Molecules of DNA taken up
1	44	64
2	66	131
3	25	30
4	19	22
5	9	10

### Co-transfection results in uptake of multiple vectors by a small proportion of parasites

**Figure 3:**
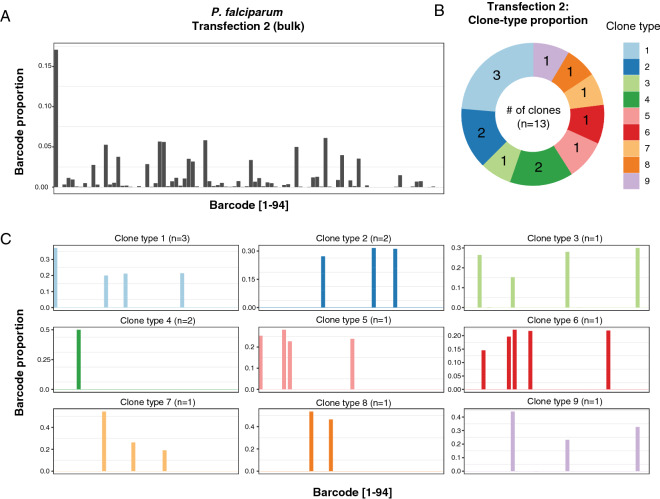
Measurement of the episome complexity in individual parasite clones obtained by limiting dilution from the most complex transfection (transfection 2 in Fig. 2). (**A**) The top panel shows the barcode complexity in the bulk population. (**B**,**C**) Barcode sequencing of 13 clones revealed nine distinct clone-types, with between 1 to 5 barcodes present per clone. The x-axis corresponds to individual barcodes, sorted by character value as opposed to numerically ordered as in Fig. 2, and the y-axis represents the proportion of each barcode in the total pool.

The fact that 9–66 vectors, from an available pool of 94, could be recovered from each transfection might suggest a potential for multiplexed screening. However, *P. falciparum* parasites are known to be able to take up at least two plasmids when they are transfected together, as evidenced by the fact that co-transfection with two different selectable markers has been used to co-localise differentially tagged proteins^20,21^, and the application of CRISPR/Cas9 to date has routinely relied on transfection with one plasmid containing the Cas9 endonuclease and another containing the homology repair construct^8^. While the efficiency of CRISPR/Cas9 editing in *P. falciparum* is notoriously variable, it does confirm that *P. falciparum* parasites can take up more than one plasmid at the same time. To assess the distribution of barcodes in the parasite population and establish the frequency with which individual parasites take up multiple plasmids, we cloned single parasites by limiting dilution from two different bulk cultures, one representing the most complex barcode mix (transfection 2) and one representing the least complex mix (transfection 5). Barcode sequencing of 13 clones from the most complex bulk culture identified 9 different clone types, each with a distinct complement of barcodes (Fig. 3a–c, Supplementary Fig. S2). However, the majority of clones contained multiple barcodes, with up to 5 barcodes detected per clone (Fig. 3c). Only 1 of the 9 clone types represented parasites with a single barcode, suggesting the majority of uptake events captured multiple plasmids. A similar result was obtained when clones were derived from the least complex bulk culture (Supplementary Fig. S1), with between 4 and 7 barcodes detected per clone. Furthermore, only three different clone types were observed from this transfection, with most of the clones having the same barcodes represented, although at different relative levels. The presence of related clone types suggests that episomes are relatively stable once captured by the parasite, as each clone was grown independently for over 1 month prior to DNA isolation and barcode sequencing.

Collectively, these observations reveal that the composition of the bulk culture can be driven by a small number of founder events, and even though transfection efficiency of *P. falciparum* is very low, there is a strong propensity for individual parasites to take up multiple plasmids.

### Dynamics of plasmid uptake is dependent on transfection method

To investigate whether transfection methodology may influence the outcome of plasmid uptake, as well as to generate a resource of fluorescent parasites for future parasite competition assays, we constructed a set of vectors for episomal expression of a range of fluorescent proteins. The fluorochromes tagBFP, MiCy (Midori-ishi Cyan) and mCherry were chosen due to their diverse excitation/emission characteristics, minimising spectral overlap (Supplementary Fig. S3a–c). These genes were cloned into expression vectors under the control of the *P. falciparum* calmodulin promoter, and with a blasticidin selection cassette in the vector backbone. The three expression plasmids were pooled in equal amounts, and used to transfect parasites using the three main transfection methodologies typically employed for *P. falciparum*. In brief, these are (i) transfection of uninfected RBCs followed by addition of purified schizonts to allow invasion of these “preloaded RBCs”^22^; (ii) transfection of ring-stage parasitized cells^23^; and (iii) schizont-stage transfection, using Percoll-purified segmented schizonts^3,24^.

**Figure 4:**
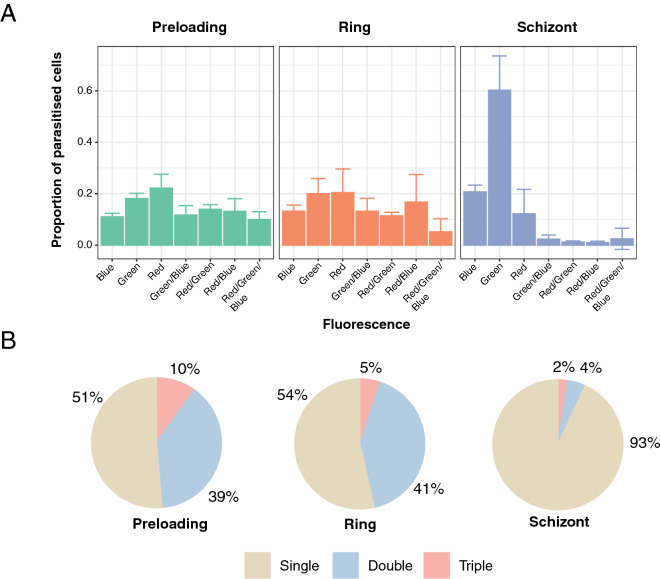
Influence of the method of transfection on plasmid uptake. (**A**) Shows proportions of each population of fluorophore-expressing parasites for each method of transfection as measured by flow cytometry. Error bars represent standard deviations derived from two to three biological replicates (see “Methods” section). (**B**) Shows the proportion of each population of parasites expressing either single, double or triple combination of fluorophores for each method of transfection. Values were rounded to the nearest percent.

The use of fluorescent proteins allowed the characteristics of plasmid uptake in each of the transfected populations to be quantified by flow cytometry (Fig. 4a,b and Supplementary Fig. S4a). Vectors were taken up in every possible combination by the population of parasites, and qualitative exploration by fluorescence microscopy identified parasites carrying one, two, and all three vectors (Supplementary Fig. S4b–e). We examined whether some of the multiply-transfected parasites may have acquired plasmids by exchange post-transfection, e.g. via microvesicle-mediated transfer^25,26^, by mixing single-fluorophore parasites that were then co-cultured for a period of 5 weeks, before quantitating fluorescence by flow cytometry. The vast majority of parasites retained their single plasmid status (Supplementary Fig. S5), suggesting that in this experimental setting the acquisition of multiple plasmids occurred during the transfection process rather than during subsequent culture.

Notably, we observed a distinct bias towards single-plasmid transfectants when using the schizont-stage approach, in contrast to both preloading and ring-stage transfection methods (Fig. 4a,b). This observation may reflect that plasmid uptake by the schizont-stage method, where free merozoites are frequently liberated from the mature schizont during the electroporation process, may occur by a different mechanism to the preloading and ring-stage approaches.

### *P. knowlesi* transfection of barcode pool yields high coverage of plasmids

Use of the schizont-stage transfection method to influence the number of plasmids captured by a single parasite may potentially solve one of the issues with library-based screening approaches in *P. falciparum*, where uptake of multiple episomes may confound the interpretation of barcode counts. However, a more fundamental limitation of current transfection approaches of *P. falciparum* remains the low transfection efficiency, with our most poorly performing transfections resulting in only 9 out of 94 barcodes represented (see Table 1). We sought to test whether a different *Plasmodium* parasite, the zoonotic species *P. knowlesi*, would display more comprehensive library representation due to its higher transfection efficiency^3^. Additional useful attributes of the *P. knowlesi* system include the ability to use the adapted line A1-H.1 that can be continuously cultured in human RBCs, and the development of CRISPR reagents for genome modification^3,27^.

**Figure 5:**
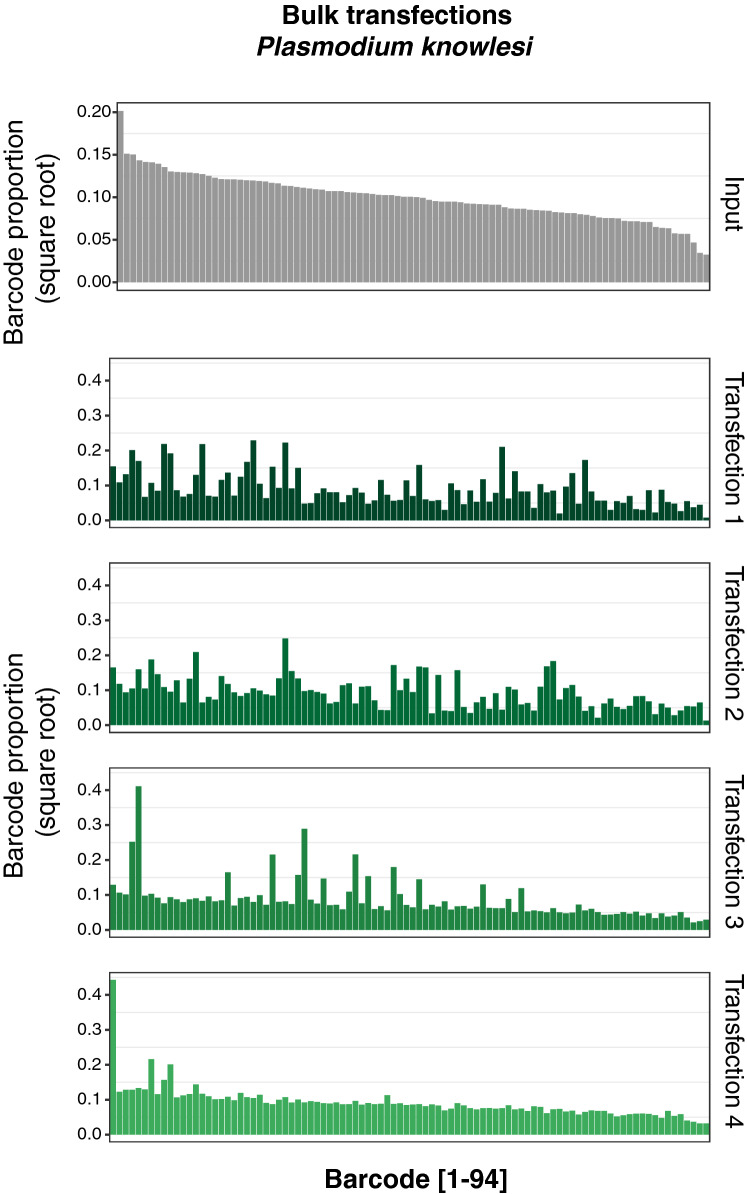
Transfection of a pool of 94 barcoded vectors into *P. knowlesi*. Individual barcodes are represented on the x-axis, and the data is sorted by the most abundant barcodes present in the input pool (first panel, grey). The y-axis represents the square-root of the proportion of each barcode in the total pool, per individual transfection as obtained by Next Generation Sequencing.

We performed four independent transfections of *P. knowlesi* parasites with the pool of 94 barcode plasmids, and selected transfectants with pyrimethamine. Parasites were observed by Giemsa-stained smears approximately 5–7 days post-transfection, and we isolated genomic DNA from the bulk cultures to perform barcode sequencing as above. In contrast to the *P. falciparum* bulk culture analysis, we observed a near-complete representation of barcodes in each of the four transfections, albeit with several fold differences in abundance (Fig. 5 and Supplementary Information). We sought to examine the distribution of barcodes in individual clones, however despite several attempts using different cloning methods, we were unable to isolate viable clonal lines, possibly due to attributes of the *P. falciparum*-based transfection vectors we employed. Nonetheless, these observations overall are consistent with the reported higher transfection efficiency of *P. knowlesi* over *P. falciparum*, and suggest this parasite may be well suited for library-based transfection experiments.

## Discussion

Experimental genetics has had a long track record of revealing new biology in *P. falciparum*, and recent developments have yielded multiple new promising tools to even more exquisitely manipulate the genome of this globally significant parasite^28^. However, all the technologies available depend on the capacity to introduce exogenous DNA into the parasite, and this remains a significant limitation for the field. Two decades after the first transfections it is still unclear what factors limit the efficiency with which DNA can be delivered to *P. falciparum* through transfection, and why this efficiency appears to be much lower than in other *Plasmodium* species^3,29^.

Our data with the complex pool of barcoded plasmids allowed us to calculate the number of DNA molecules delivered per transfection. We estimate that ~ 6 × 10^7^ parasites were placed in the transfection cuvette, and collectively took up between 10 and 131 molecules of DNA upon electroporation for a maximum transfection efficiency of ~ 2 × 10^–6^, assuming independent plasmid uptake modelled under a Poisson distribution. This underlines the very low transfection efficiency of *P. falciparum* in contrast with *P. knowlesi* or *Toxoplasma gondii*^3,30^. Notably, transfection of the same barcode pool into *P. knowlesi* yielded a near complete and relatively homogenous representation of these plasmids, without being dominated by a small subset of barcodes as observed in the *P. falciparum* transfections. While we observed a linear relationship between the abundance of individual barcodes in the pool and their abundance in each of the *P. knowlesi* transfections, *P. falciparum* displayed a tendency for barcodes to be taken up in a more stochastic manner (Supplementary Information).

The data also yields another unexpected hurdle to large-scale screening in *P. falciparum.* If each of these 10–131 uptake events were independent, one would expect each of these molecules to be found in a different parasite. Under this model of independent uptake events, the chances of a single parasite taking up 2 molecules would be ~ 4 × 10^–12^; and given that there are only ~ 6 × 10^7^ parasites in the cuvette, we would never expect to see such a cell. In fact, our data from clonal dilution and fluorescence experiments suggest that, depending on the transfection methodology, the majority of transfection events involve the uptake of multiple molecules despite there being no selective pressure to maintain multiple distinct plasmids, and more than expected by chance. This phenomenon is driven by the uptake of multiple plasmids during transfection, rather than the exchange of plasmids between parasites post-transfection, for example through microvesicles^25,26^, as we did not see exchange of plasmids in our experimental context.

To better understand the parameters that might affect plasmid uptake, we tested the impact of different transfection approaches, comparing three standard methods used by the community. Preloading of RBCs involves transfection of uninfected RBCs that are then invaded by parasites, which then spontaneously take up plasmid DNA by an undefined mechanism^22^. Transfection of ring-stage parasite cultures was the first method developed for *P. falciparum*^23,31^. More recently, the schizont-stage transfection approach has become more widely used, and entails Nucleofector-based electroporation of segmented schizonts containing mature merozoites^3,24^. We observed that the propensity of parasites to take up multiple plasmids was similar with both the preloading and ring-stage transfection methods, possibly as both approaches may ultimately deliver DNA into the parasite by a similar mechanism. In contrast, the schizont-stage method yielded the highest proportion of single-plasmid transfectants, which we speculate may occur through direct electroporation and transient pore formation of free merozoites.

There are a number of possible bottlenecks in *Plasmodium* transfection. Our data suggest that availability of DNA is not likely to be a crucial limitation. Furthermore, the use of "two plasmid" systems, for instance for supplying a donor and a Cas9 vector, is unlikely to lead to significantly lower efficiencies than one plasmid systems with current preloading or ring-stage transfection conditions. The overall higher transfection efficiency of schizont-stage transfection may overcome any limitation in multiple plasmid uptake if the appropriate selective pressure is provided. A previous similar analysis carried out in Jurkat cells using fluorescent markers provided a result much closer to a Poissonian process, with cells differing in their susceptibility to transfection by at most a factor of three^32^. One potential explanation for our data is that for the preloading and ring-stage transfection approaches, only a few *P. falciparum* cells within a culture are actually competent to take up plasmids, and do so by a non-specific ‘bulk flow’ process that often results in uptake of multiple molecules. Efforts to identify the nature of these rare parasites or uptake events, and also what distinguishes *P. falciparum* from other *Plasmodium* species with much higher transfection efficiencies may yield insights^3,29^. Notably, transfection of *P. berghei* and *P. knowlesi* has predominantly employed the schizont-stage transfection approach, and our observation that this method enriches for single-plasmid uptake suggests it may work by a different mechanism to transfection of other stages. Further understanding these dynamics and the factors that control them will be particularly important for the development of multiplexed library-based screens, including overexpression, CRISPR-interference or CRISPR-activation approaches, where low multiplicities of transfection will be crucial. This knowledge will be fundamental to the exploration of the parasite’s genome and the unravelling of gene function as well as host-parasite interactions.

## Methods

### Generating fluorochrome-expressing vectors

Midori-ishi Cyan (acquired from Addgene), tagBFP and mCherry sequences were amplified by PCR using specific primers with restriction sites for AvrII for the forward and XhoI for the reverse primers. The fragments were ligated into the pDC2 *Plasmodium* expression vector^33^ containing a PfCAM promoter and a Blasticidin-S deaminase selectable marker, for drug selection at 2 µg/mL Blasticidin.

### Generation of library of barcoded plasmids

The templates for barcode amplification were derived from PlasmoGEM resource pJazz vectors (https://plasmogem.sanger.ac.uk), provided as glycerol stocks. 96 different bacterial stocks each possessing an individual barcode vector were incubated individually in deep 96-well plates (maximum volume 2 mL) overnight at 30 °C, in TB medium with 30 µg/mL of kanamycin. After each bacterial culture reached an appropriate optical density, and assuming similar growth rates between them, the cultures were pooled and the library of 96 vectors was extracted using a Macherey Nagel midi prep kit. A 120bp barcode amplicon was amplified directly from the pool using primers p212 (CAATTAATGATGTATACCGCCTTCAATTTCGATGGGTAC) and p219 (CTAAGAAGGTTATAGAGGCGTAATTCGTGCGCGTCAG), which contained sequence overlap to a NheI/NcoI digested *P. falciparum* vector pCC1 to permit Gibson assembly (Fig. 1a). The final assembled vector containing all possible 96 barcodes cloned into pCC1 was transformed into One-Shot TOP10 competent *E. coli* and plated onto LB plates with ampicillin. Colonies were pooled into a large culture and a midiprep was performed to generate the transfection pool.

### Parasite cultures

Parasites were propagated at 37 °C in standard parasite culture media essentially as described^34^ and with a gas mix of 1% O_2_, 3% CO_2_ and 96% N_2_. All parasite strains were routinely cultured in O + Red Blood Cells (RBCs) provided by anonymous healthy donors from National Health Services Blood and Transplant (NHSBT). Informed consent from donors was obtained by NHSBT as part of their recruitment process, and the use of RBCs from human donors was performed in accordance with relevant guidelines and regulations, with approval from the NHS Cambridgeshire Research Ethics Committee and the Wellcome Sanger Institute Human Materials and Data Management Committee. Synchronization of cultures was performed using 5% sorbitol in water^35^.

### Transfection of parasites

For pooled barcode transfections in *P. falciparum*, plasmid DNA (50 µg per pool) was resuspended in 100 µL of buffer P3 (Lonza) with 4 µL ATP (625 mM). Cultures containing high parasitemia (approximately 10%) at mostly ring stage were centrifuged and 100 µL of packed RBCs per transfection were washed with cold cytomix, resuspended in P3 buffer with DNA and ATP and electroporated with a Lonza Nucleofector 4D using the programme P3/CM-150. For pooled barcode transfections in *P. knowlesi,* segmented schizonts were purified on a Nycodenz cushion. 30 µL of packed schizonts were mixed with 90 µL of plasmid DNA (corresponding to 25 µg diluted in P3 buffer) and electroporated with the Lonza Nucleofector 4D using the programme P3/FP158. Electroporated schizonts were mixed with 150 µL of packed RBCS in 650 µL of complete media and incubated on a thermomixer at 37ºC for 30–45 min before being transferred to a culture flask for continuous culture under pyrimethamine (100 µM) pressure.

For transfection of the fluorochrome vectors into *P. falciparum*, 20 µg of each construct were co-transfected in Dd2 parasites using the following procedures:

- For schizont-stage transfections, parasites were tightly synchronised using 2 to 3 rounds of Percoll/sorbitol purification. On the day of transfection, late-stage schizonts were purified on a 63% Percoll cushion and treated with the PKG inhibitor compound 2 at 1.5 µM in complete media for 2 h in order to enrich for segmented schizonts^36^. Egress blockade was released by washing out compound 2 and incubating parasites in complete media for 15 min. 20 µL of packed segmented schizonts were then mixed with 100 µL of plasmid DNA in P3 buffer and electroporated with the Lonza Nucleofector 4D using the programme P3/FP158. Parasites were then transferred to culture flasks in complete media with RBCs at 10% hematocrit and shaken at 37ºC for 20 min. Culture was then diluted to 3% hematocrit and the following day parasites were placed under continuous drug pressure with 2 µg/mL Blasticidin.
- Pre-loading transfections were performed as described^22^. Briefly, 300 µL of packed fresh RBCs were mixed with 150 µL of plasmid DNA diluted in cytomix and electroporated using a BioRad Gene Pulser Xcell. Cells were left to recover for 30 min at 37 °C, washed and mixed with purified segmented schizonts. Continuous drug pressure with 2 µg/mL Blasticidin was applied 48 h later.
- For ring stage transfections, purified segmented schizonts were left to reinvade fresh RBCs for 2–3 h and early-stage rings were transfected with 50 µg plasmid DNA using the BioRad Gene Pulser Xcell following the standard protocol^23^. Continuous drug pressure with 2 µg/mL Blasticidin was applied the following day.

In total, three independent schizont-stage transfections were carried out, while ring-stage and pre-loading transfections were performed in duplicate. The preloading transfections were split into two the day after parasite reinvasion, such that 4 cultures were ultimately analysed.

### Library preparation for next generation sequencing

Genomic DNA was extracted using a Qiagen Blood and Tissue Kit after parasites reached 5% parasitaemia. Once gDNA was extracted and measured, a nested PCR reaction targeting the constant flanking regions of the barcode was performed. 50 ng of gDNA was used for amplification using p1356 (TCGGCATTCCTGCTGAACCGCTCTTCCGATCTGTAATTCGTGCGCGTCAG) and p1357 (ACACTCTTTCCCTACACGACGCTCTTCCGATCTCCTTCAATTTCGATGGGTAC) containing adapters for Illumina. Paired-end index primers (Illumina Nextera) followed on a secondary PCR. All reactions were performed using a 2X KAPA Hot-Start Master Mix (Kapa Biosciences). A PCR purification step was performed only on the second PCR using a Macherey Nagel PCR purification kit and eluted DNA was measured, multiplexed and diluted to a final concentration of 4 nM. Samples were loaded onto an Illumina MiSeq sequencer, using a MiSeq Reagent Kit v2 (300 cycle). They were loaded at a low cluster density (< 400 k cluster density), and 50% of PhiX was spiked in, as described in Gomes et al. for low complexity libraries^19^.

### Barcode counting and analysis

Barcode counting was performed as described^19^. Briefly, raw reads obtained from the Illumina MiSeq, represented by unique index tags, were separated and analysed with a script that identified correct flanking sequences and counted exact matches of unique barcodes between these constant regions. Barcode counts for all experiments are provided in the Supplementary Data.

### Cloning of parasites by limiting dilution

To clone transfected parasites, we used limiting dilution cloning as described^37^ with the following modifications. Parasites were diluted into a 96-well plate at 0.5–0.8 parasites per well at 1.8% haematocrit, expecting approximately 50% of the plate to contain clonal parasites. Detection of positive wells was first performed using the DNA stain SYBR Green, discarding either empty wells or those with much higher fluorescence than the average, indicating possibly more than one parasite was inoculated at seeding.

### Flow cytometry

Distribution of the fluorophore-expressing plasmids among transfected parasites was analysed on multiple days from 35 days post-transfection, once all cultures had reached a parasitaemia of 1% or above. Parasitized red blood cells were diluted to 0.15% haematocrit in PBS and examined on a CytoFLEX 5 (Beckman Coulter) using the following excitation/emission values: 405/450 nm for TagBFP, 488/530 nm for miCyan and 488/610 nm for mCherry. 100,000 events were recorded per sample. Uninfected red blood cells and untransfected parasite cultures were used as controls for gating and selecting for singlets. FlowJo (7.6.5) was used for the analysis; the gating strategy is described in Supplementary Fig. S4a.

### Microscopy

Standard blood smear microscopy was performed to determine parasitemia. In brief, a small aliquot of culture was smeared on a glass slide, fixed with 100% methanol and stained with a 10% Giemsa solution (Sigma-Aldrich). Fluorescent parasites were imaged on a Leica TCS SP8 confocal microscope (Leica Microsystems). Schizonts of each transfected culture were purified on a 63% percoll gradient and fixed with fresh 4% paraformaldehyde for 20 min. The schizonts were placed on slides, dried and mounted with ProLong Diamond antifade mountant (Thermo Fisher) overnight at 4 °C.

## Supplementary information

Supplementary file1

Supplementary file2 (XLSX 43 kb)

## Data Availability

All data necessary to perform the analysis of the barcode sequencing of transfections are available (Supplementary Data), as well as the data on the barcode complexity of the bulk culture and isolated clones shown in (Figs. 2, 3, 5, Supplementary Figs. S1, S2).
