# Supplementary material for: Defining multiplicity of vector uptake in transfected Plasmodium parasites
Source: Sci Rep. 2020 Jul 2;10:10894. doi: 10.1038/s41598-020-67791-z (PMC7331667; doi:10.1038/s41598-020-67791-z)
Supplement: Supplementary file 1 — Supplementary file1 [file 41598_2020_67791_MOESM1_ESM.pdf]

Supplementary information

Defining multiplicity of vector uptake in transfected *Plasmodium* parasites

Manuela Carrasquilla[1,2,#], Sophie Adjalley[1,#], Theo Sanderson[1,3], Alejandro Marin-Menendez[1,4], Rachael Coyle[1], Ruddy Montandon[1,5], Julian C. Rayner[1,6], Alena Pance[1], Marcus C. S. Lee[1]

[1] Wellcome Sanger Institute, Wellcome Genome Campus, Hinxton, UK.

[2] Department of Immunology and Infectious Diseases, Harvard T.H. Chan School of Public Health, Boston, USA.

[3] The Francis Crick Institute, London, UK.

[4] MIVEGEC, IRD, CNRS, University of Montpellier, Montpellier, France.

[5] Wellcome Centre for Human Genetics, Oxford, UK.

[6] Cambridge Institute for Medical Research, Cambridge, UK.

[#] These authors contributed equally to this study.

Supplementary Figure 1 page 2
Supplementary Figure 2 page 3
Supplementary Figure 3 page 4
Supplementary Figure 4 page 5
Supplementary Figure 5 page 6
Analysis of episomal barcode distributions in *P. falciparum* page 7
Analysis of episomal barcode distributions in *P. knowlesi* page 13

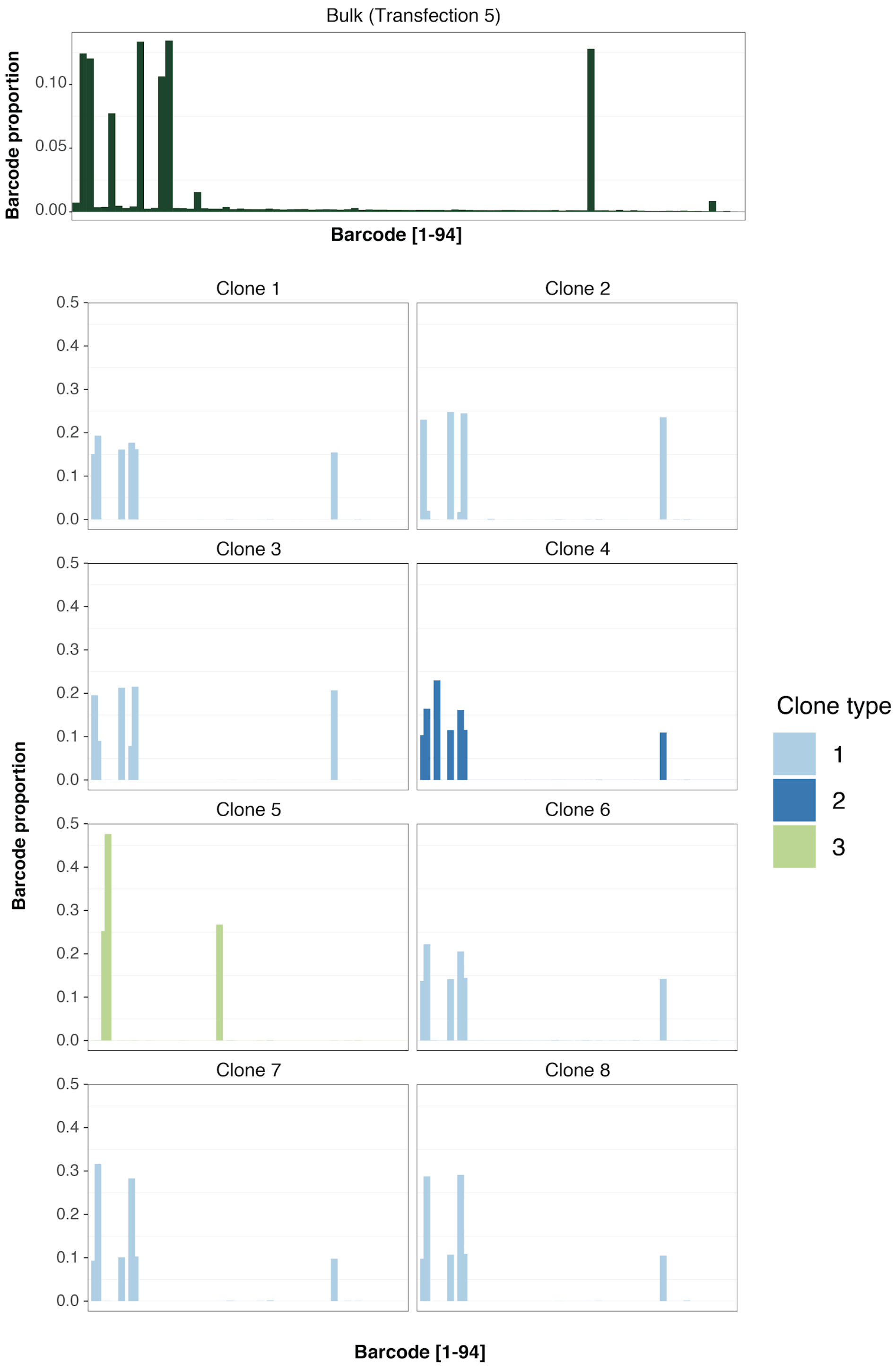

Supplementary Figure 1. Complexity of episomes in clonal parasites obtained from transfection 5. Measurement of the complexity of episomes in individual parasite clones obtained by limiting dilution from the least complex bulk culture (transfection 5 in Fig. 2).

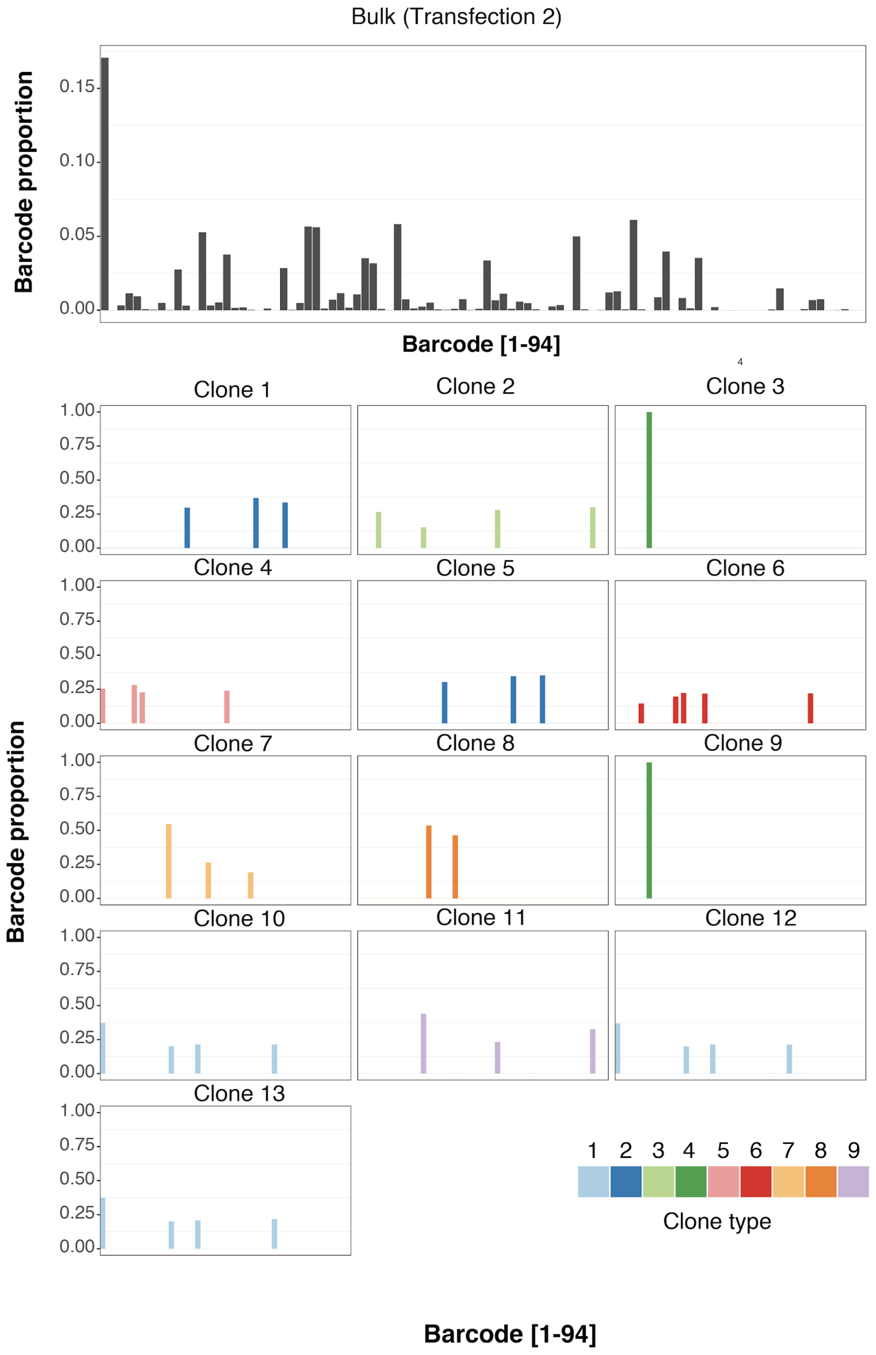

Supplementary Figure 2. Complexity of episomes in clonal parasites obtained from transfection 2. Measurement of the complexity of episomes in individual parasite clones obtained by limiting dilution from the most complex bulk culture (transfection 2 in Fig. 2).

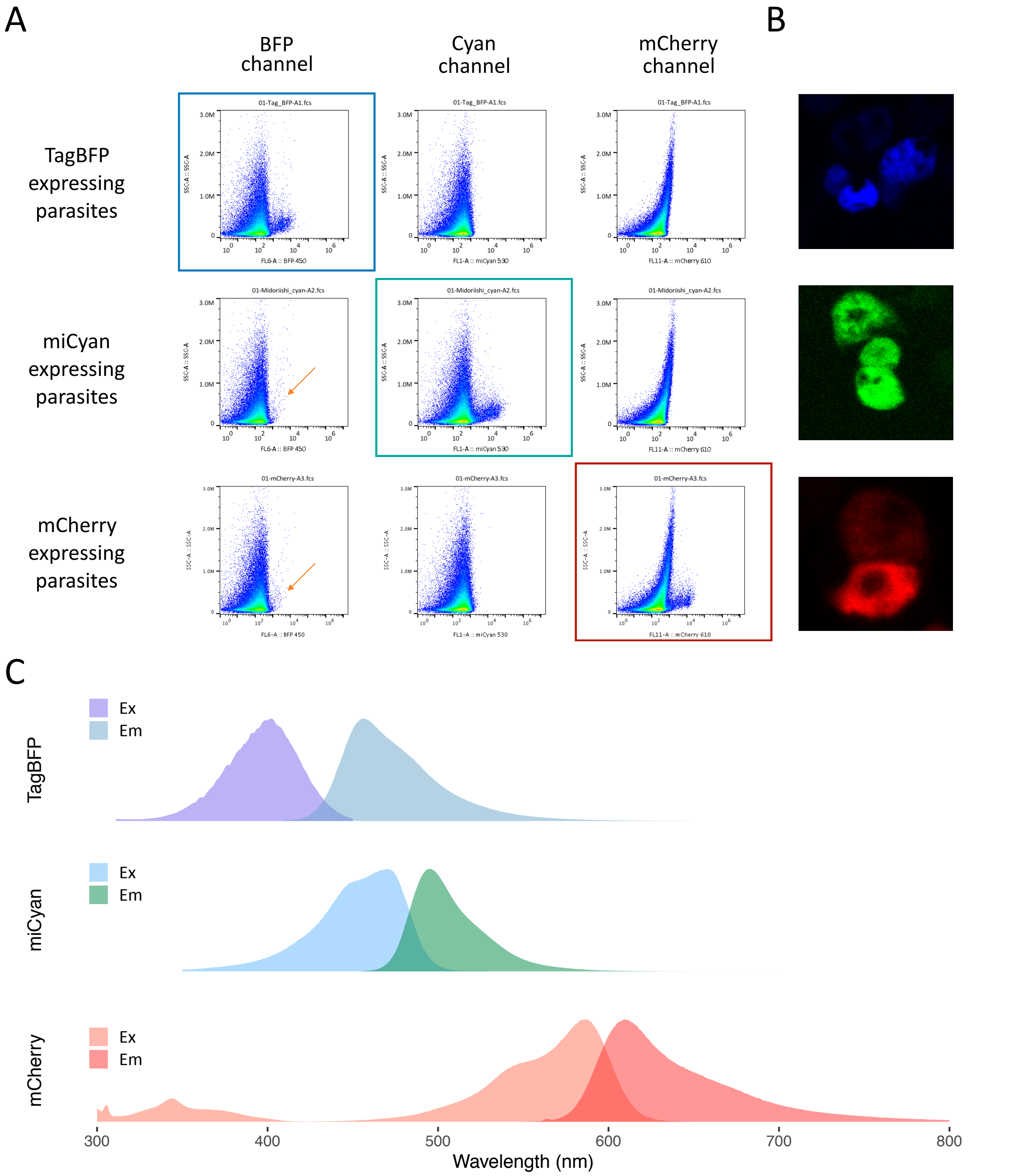

Supplementary Figure 3. Assessment of parasites transfected with individual fluorescent reporter plasmids. **A**. Flow cytometry analysis shows that parasites expressing each fluorophore individually are detected in the expected channel (colored squares). Note that a minority of TagBFP-expressing parasites are detected in both miCyan and mCherry channels (red arrows). **B**. Fluorescent microscopy images of blue, green and red parasites expressing individual fluorophores, TagBFP, miCyan and mCherry, respectively. **C**. Excitation (Ex) and Emission (Ex) spectra of each fluorophore.

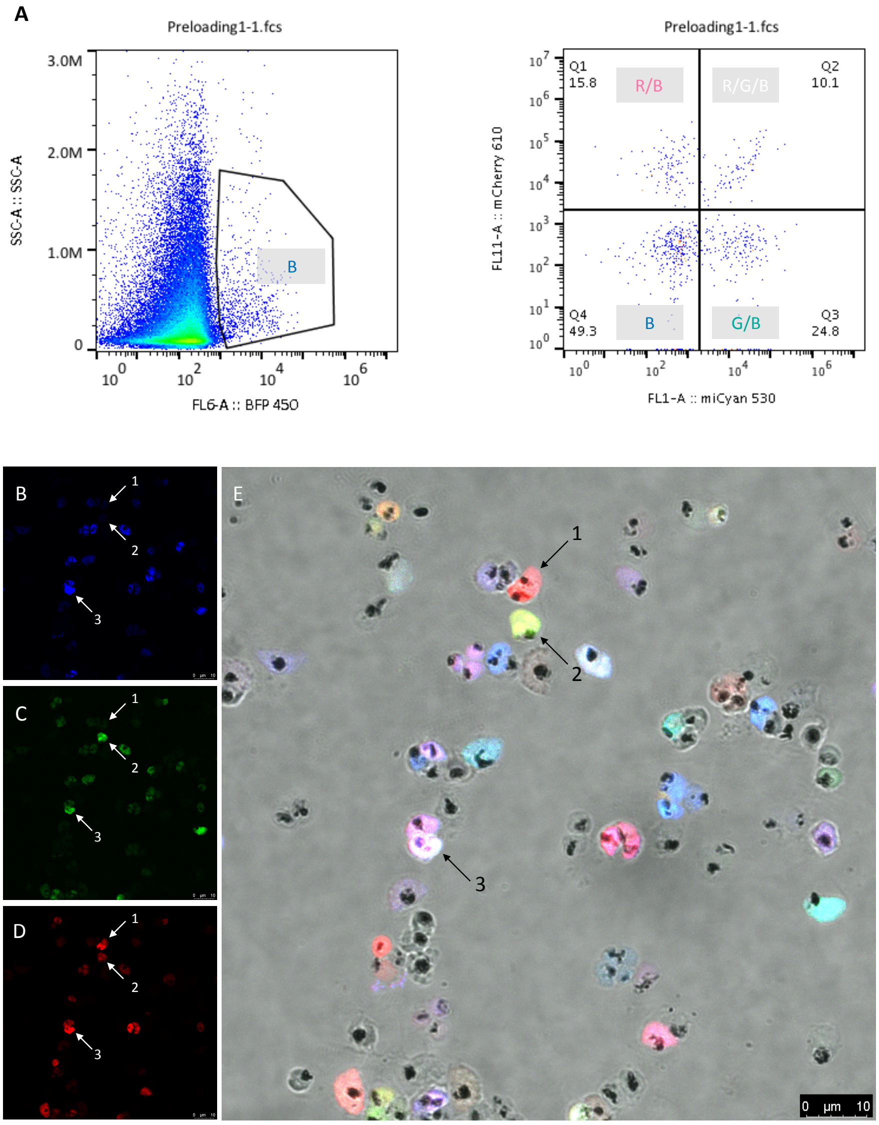

Supplementary Figure 4. A. Representative example illustrating the gating strategy for the flow cytometry assessment of single-, double-, and triple-fluorescent parasites from a transfection using the pre-loading approach. The population of parasites expressing tagBFP (labelled "B") is identified using the Side Scatter parameter (SSC) in the BFP450 channel (left panel). This population of parasites is then assessed for expression of miCyan (labelled "G") and/or mCherry (labelled "R") by analyzing its distribution in the two other channels (right panel). This allows for the quantification of parasites expressing either only TagBFP("B"), mCherry and TagBFP ("R/B"), miCyan and TagBFP ("G/B") or all three fluorophores ("R/G/B"). **B-E.** Fluorescence microscopy of a ring-stage transfection, showing individual channels for (B) tagBFP, (C) miCyan, (D) mCherry, as well as (E) a merged image including brightfield. Arrows label exemplar parasites simultaneously expressing one (1), two (2) or three fluorochromes (3), respectively.

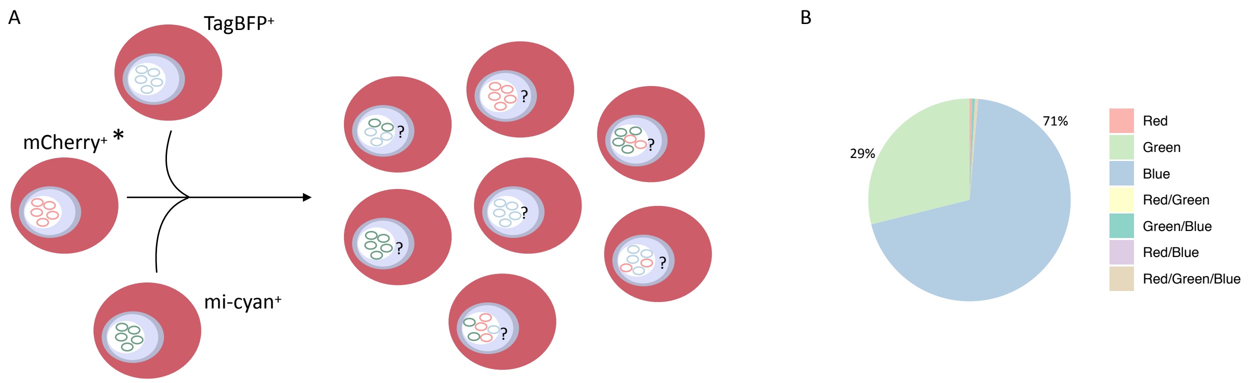

Supplementary Figure 5. A. Co-culture of a mixture of single-fluorophore parasites was performed over a period of 5 weeks, and the bulk culture was analysed by flow cytometry. *Note that the mCherry-expressing parasites grew poorly initially and were therefore depleted from the final co-culture. Experiment was performed in three independent replicates. **B**. No appreciable plasmid exchange was observed, with the majority of parasites containing episomes expressing a single fluorophore, mostly of the blue (TagBFP) or green (miCyan) type. Values were rounded to the nearest percent.

Analysis of episomal barcodes distributions (Plasmodium falciparum)

We are first going to fit distributions to estimate the number of unique barcodes in each transfection. First we load the data:

```
library(knitr)
library(kableExtra)
library(tidyverse)
library(mixtools)
data<-read_csv("Pf_BulkTransfections.csv")
data$Barcode_number<-NULL
colnames(data)<-gsub("Barcode_sequence","barcode",colnames(data))

narrow<- data %>% gather("type","val",-barcode)
```

```
ggplot(narrow,aes(x=barcode,y=val))+facet_wrap(~type) +geom_bar(stat="identity")+
  labs(y="Barcode counts",x="Barcode")+theme(axis.text.x=element_blank(),axis.ticks.x=element_blank())+[illegible]
```

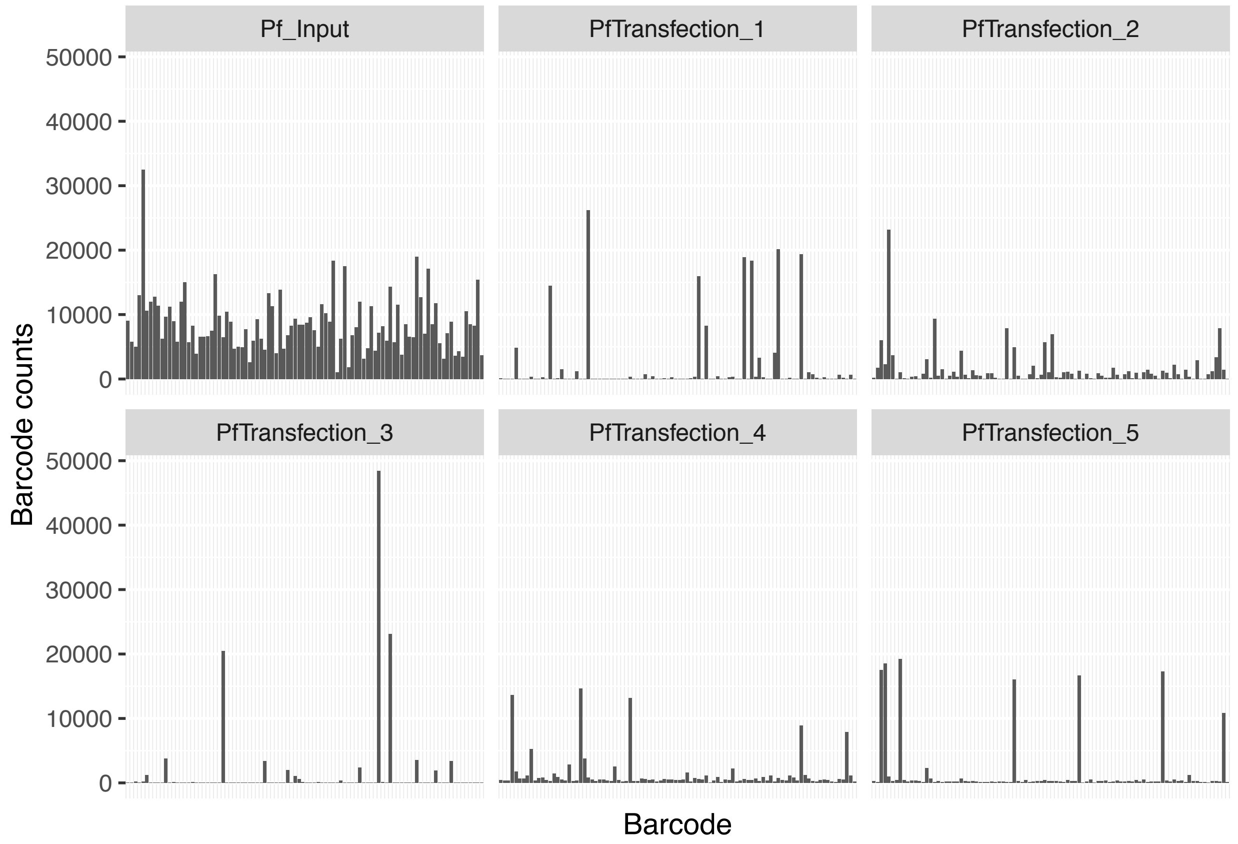

Now we convert everything to log2 format, and work out for each barcode the difference between its value in the input and its value in each of the transfections. We plot the distribution of these differences.

```
narrowlog <- mutate(narrow,val=log2(val+0.5))
input<-filter(narrowlog,type=="Pf_Input")
noninput<-filter(narrowlog,type!="Pf_Input")
both<-inner_join(noninput,input,by="barcode") %>% mutate(diff=val.x-val.y)
```

```r
colnames(both)<-gsub("type.x","Transfection",colnames(both))

ggplot(both,aes(x=val.y,y=val.x,color=Transfection))+geom_point(size=1) +facet_wrap(~Transfection)+labs
```

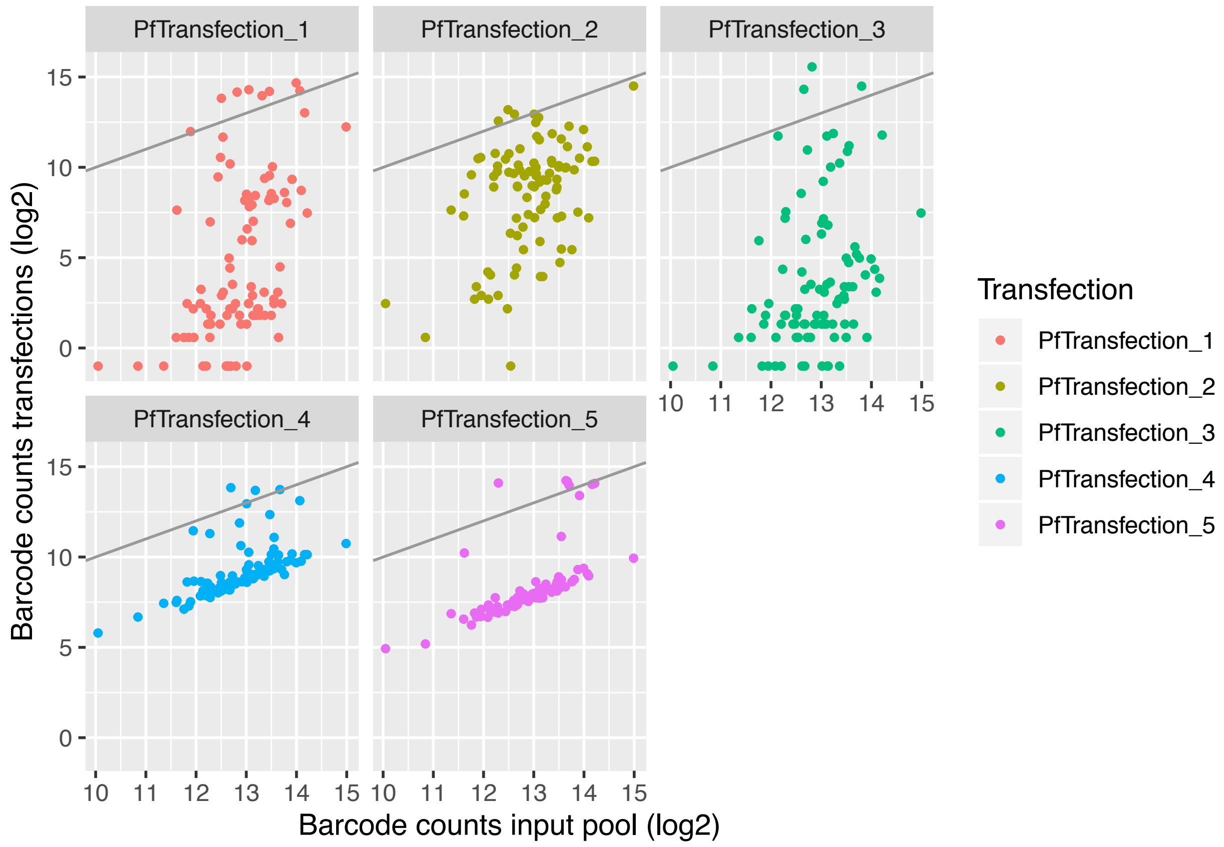


```r
ggplot(both,aes(x=diff,color=Transfection))+geom_density() +labs(x="Difference (in log-base-2)",y="Dens
```

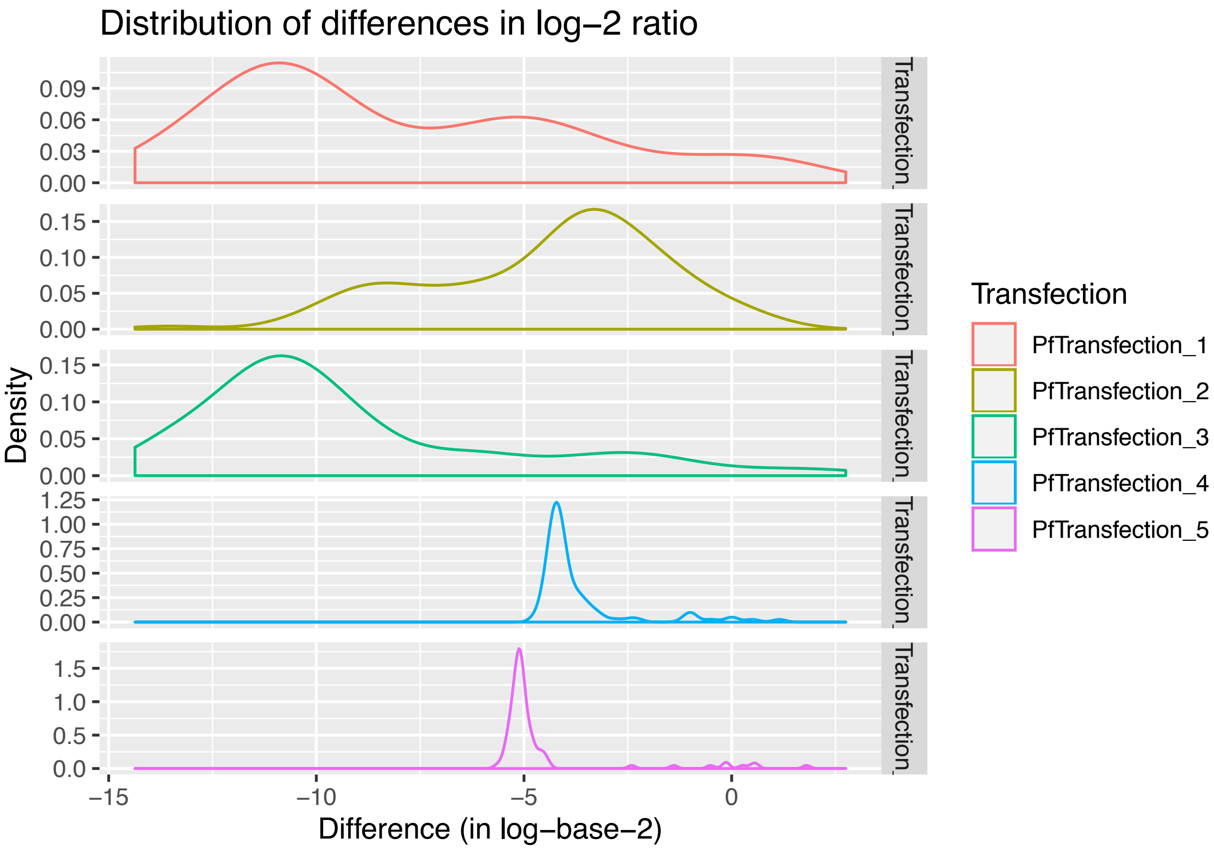

Since all barcodes should have equal fitness, variance in this plot should arguably only be explained by two factors: stochasticity in whether a barcode ever made it into the population of parasites, and then stochasticity in the growth of those parasites. There appears to be a bimodal distribution.

We model this as the sum of two normal distributions, in the expectation that the more distribution with the low mean represents complete failure to establish a transfectant and the more positive distribution represents establishment of a transfectant. We deconvolute this mixture below.

```
dnormWithLambda<-function(x,mean,sd,lambda){
  results<-dnorm(x,mean,sd)
  return(results*lambda)
}
splitUp <- function(data){
  mod<-normalmixEM(data$diff,epsilon=1e-20,maxrestarts=500)

  lambdas=mod$lambda
  mus=mod$mu
  sigmas=mod$sigma

  if (mus[1]>mus[2])
  {
    lambdas=rev(lambdas)
    mus=rev(mus)
    sigmas=rev(sigmas)

  }
p<-ggplot(data,aes(x=diff))+geom_density(color="red")+stat_function(color="blue",linetype=2,fun = dnormW
 print(p)
```

```
        return(data.frame(lambda=lambdas[2]))

}

library(tidyverse)
resultsFirst<-both %>% group_by(Transfection) %>% do(splitUp(.)) %>% dplyr::mutate(numberOfBarcodes=rou
```

```
## number of iterations= 45
```

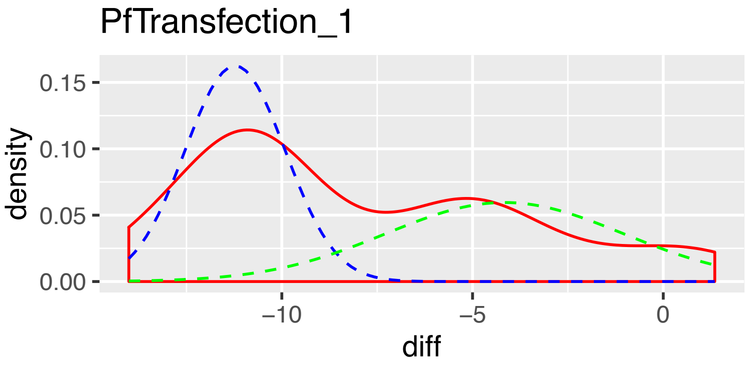


```
## number of iterations= 118
```

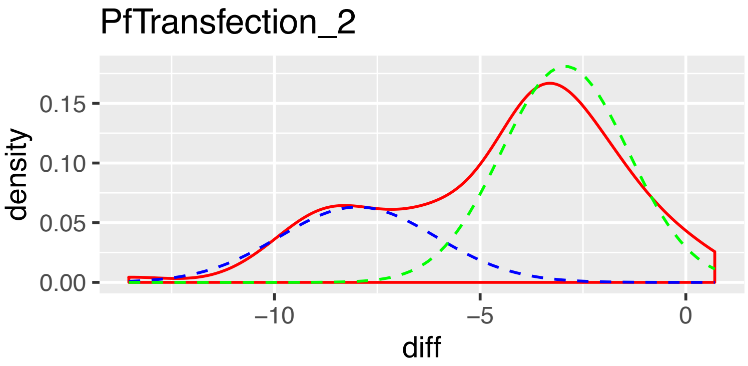


```
## number of iterations= 75
```

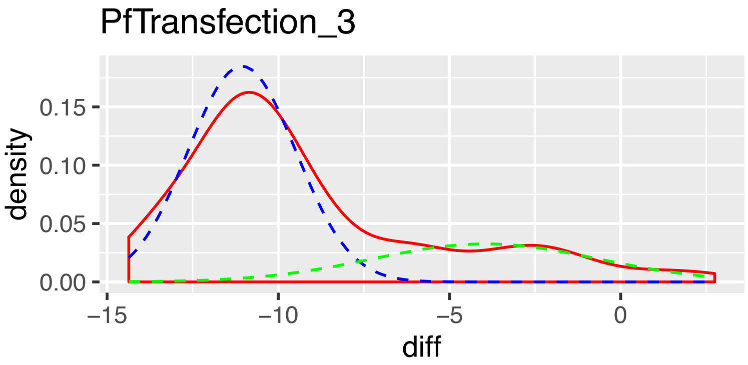


```
## number of iterations= 71
```

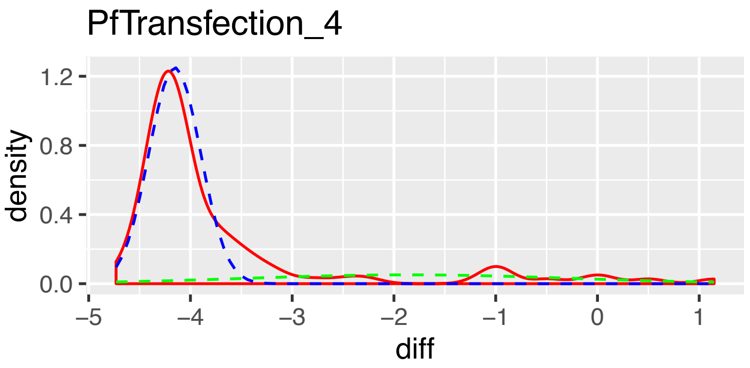


```
## number of iterations= 8
```

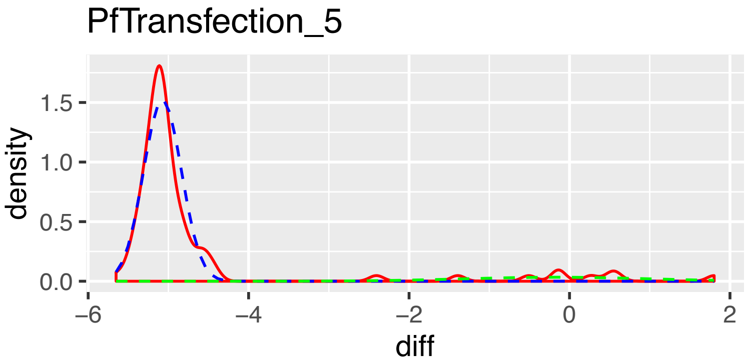


```
resultsFirstDisp <- resultsFirst
resultsFirst
```

```
## # A tibble: 5 x 3
## # Groups:   Transfection [5]
##   Transfection     lambda numberOfBarcodes
##   <chr>             <dbl>            <dbl>
## 1 PfTransfection_1 0.464                44
## 2 PfTransfection_2 0.699                66
## 3 PfTransfection_3 0.269                25
## 4 PfTransfection_4 0.204                19
## 5 PfTransfection_5 0.0958                9
```

```
colnames(resultsFirstDisp)=c("Transfection","Lambda","Unique Barcodes")
kable(resultsFirstDisp,format="latex",booktabs=T)
```

Transfection	Lambda	Unique Barcodes
PfTransfection_1	0.4641849	44
PfTransfection_2	0.6994109	66
PfTransfection_3	0.2691667	25
PfTransfection_4	0.2038328	19
PfTransfection_5	0.0957712	9

We now have an estimate of the number of unique barcodes in each transfection.

We can now ask another question. Given the proportion of various barcodes in the input, if we simulate a certain number of barcodes being taken up by parasites, then how many distinct barcodes would we expect to recover (given that one batcode might be taken up more than once)?

```
input2<- narrow %>% filter(type=="Pf_Input") %>% mutate(prop=val/sum(val))
simulate <- function(numberTakenUp){
```

```
answer=median(replicate(100,length(unique(sample(x = 1:nrow(input), numberTakenUp, replace = T, prob = 
return(answer)
}
numberTakenUp<-1:500
numberOfBarcodesRecovered<-sapply(numberTakenUp,simulate)
df<-data.frame(numberTakenUp=numberTakenUp,numberOfBarcodesRecovered=numberOfBarcodesRecovered)

ggplot(df,aes(x=numberTakenUp,y=numberOfBarcodesRecovered))+geom_line()+labs(x="Number of molecules tak
```

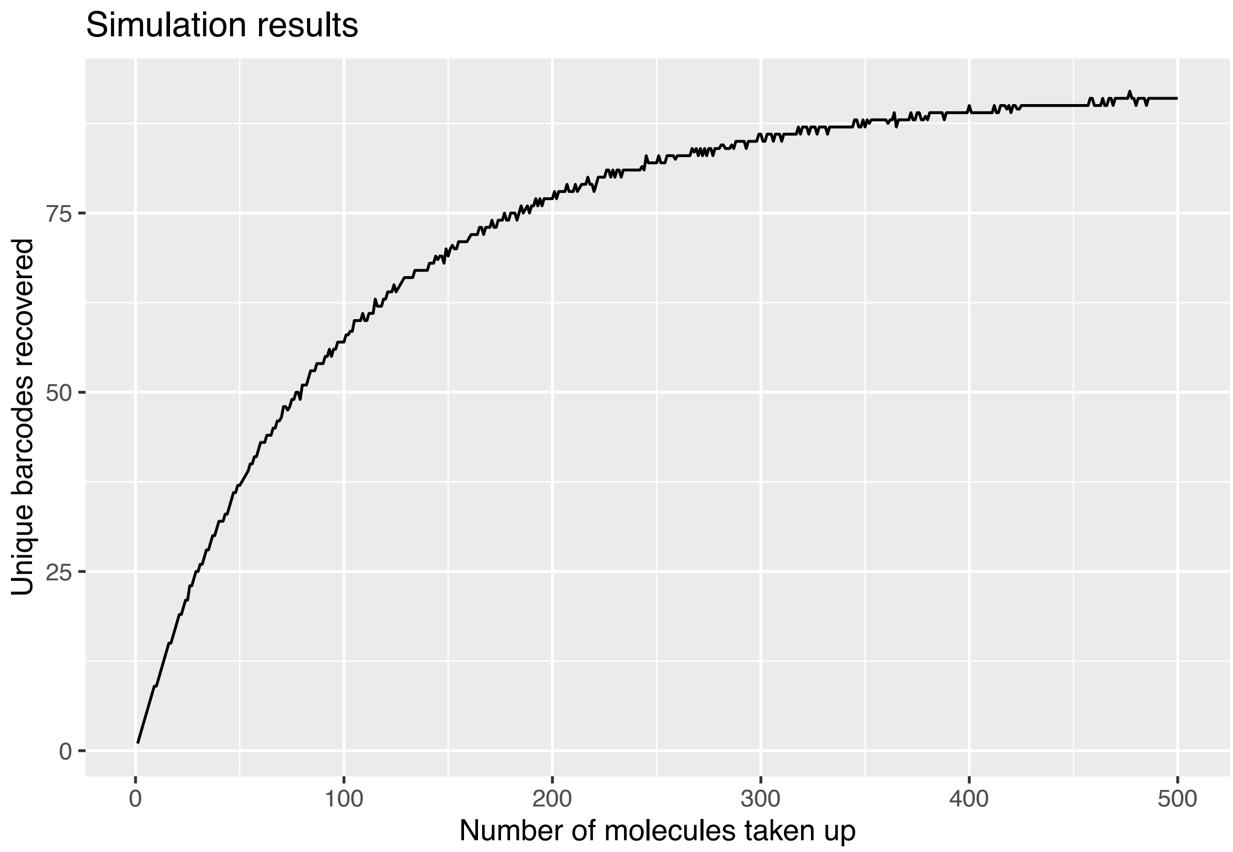


```
df <- df %>% group_by(numberOfBarcodesRecovered) %>% summarise(numberTakenUp=mean(numberTakenUp))
```

We can now use this data to estimate how many molecules of DNA were taken up in each transfection.

```
combination<-inner_join(resultsFirst,df,by=c("numberOfBarcodes"="numberOfBarcodesRecovered"))
colnames(combination)=c("Transfection","Lambda","Unique Barcodes","Molecules of DNA taken up")
kable(combination,format="latex",booktabs=T)
```

Transfection	Lambda	Unique Barcodes	Molecules of DNA taken up
PfTransfection_1	0.4641849	44	64.0
PfTransfection_2	0.6994109	66	131.0
PfTransfection_3	0.2691667	25	29.5
PfTransfection_4	0.2038328	19	21.5
PfTransfection_5	0.0957712	9	9.5

We conclude that in these transfections parasites took up and stabilised 9-130 DNA molecules, depending on the transfection.

Analysis of episomal barcodes distributions (Plasmodium knowlesi)

We are first going to fit distributions to estimate the number of unique barcodes in each transfection. First we load the data:

```
library(knitr)
library(kableExtra)
library(tidyverse)
library(mixtools)
```

```
data <- read_csv("Pk_BulkTransfections.csv")
```

```
## Parsed with column specification:
## cols(
##   Barcode_number = col_double(),
##   Barcode_sequence = col_character(),
##   Pk_Input = col_double(),
##   PkTransfection_1 = col_double(),
##   PkTransfection_2 = col_double(),
##   PkTransfection_3 = col_double(),
##   PkTransfection_4 = col_double()
## )
```

```
data$Barcode_number<-NULL
colnames(data)<-gsub("Barcode_sequence","barcode",colnames(data))

narrow<-data %>% gather("type","val",-barcode)
narrow<- filter(narrow, barcode !="discarded")
```

```
ggplot(narrow,aes(x=barcode,y=val))+facet_wrap(~type) +geom_bar(stat="identity")+
  labs(y="Barcode counts",x="Barcode")+theme(axis.text.x=element_blank(),axis.ticks.x=element_blank())+[illegible]
```

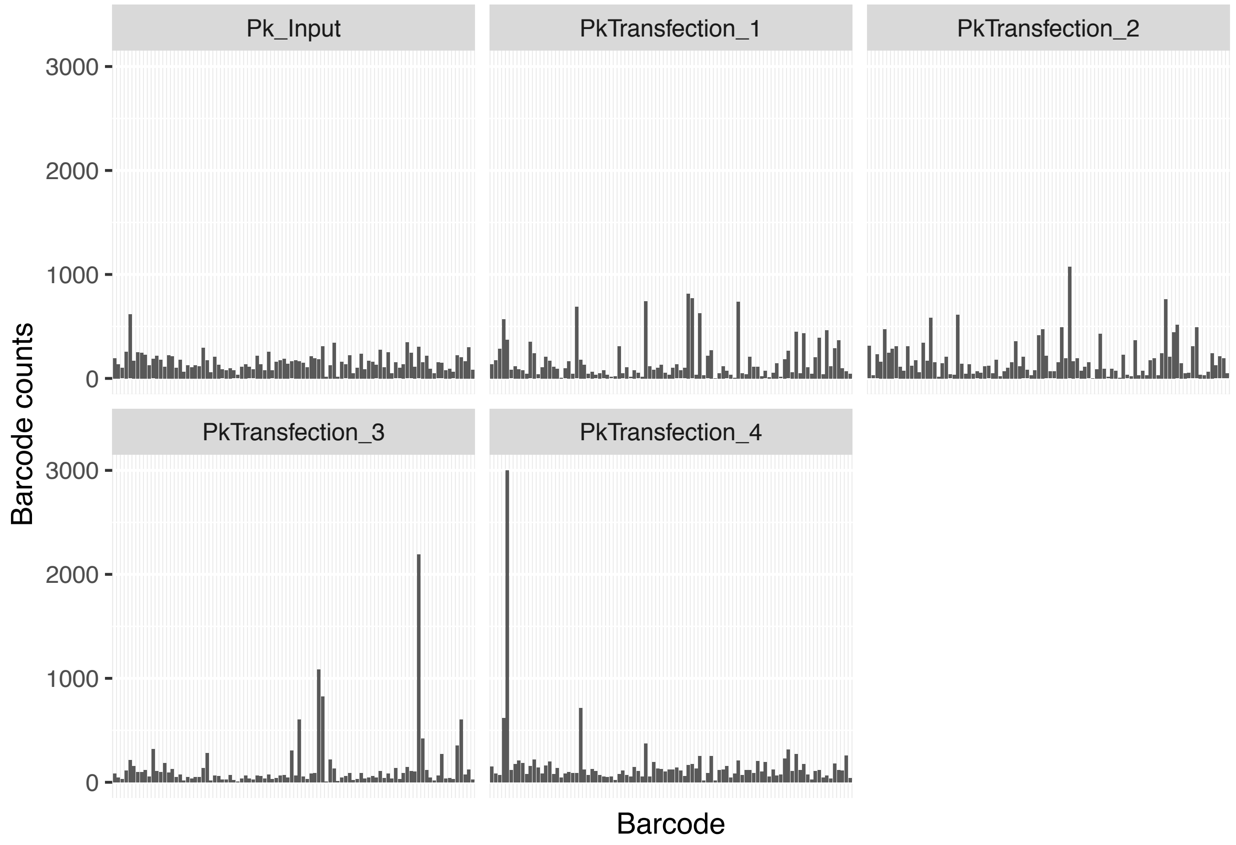

Now we convert everything to log2 format, and work out for each barcode the difference between its value in the input and its value in each of the transfections. We plot the distribution of these differences.

```
narrowlog <- mutate(narrow,val=log2(val+0.5))
narrowlog <- filter(narrowlog, barcode !="discarded")
input<-filter(narrowlog,type=="Pk_Input")
noninput<-filter(narrowlog,type!="Pk_Input")
both<-inner_join(noninput,input,by="barcode") %>% mutate(diff=val.x-val.y)

colnames(both)<-gsub("type.x", "Transfection",colnames(both))

ggplot(both,aes(x=val.y,y=val.x,color=Transfection))+geom_point(size=1) +facet_wrap(~Transfection)+labs
  geom_abline(slope = 1,color="#999999")
```

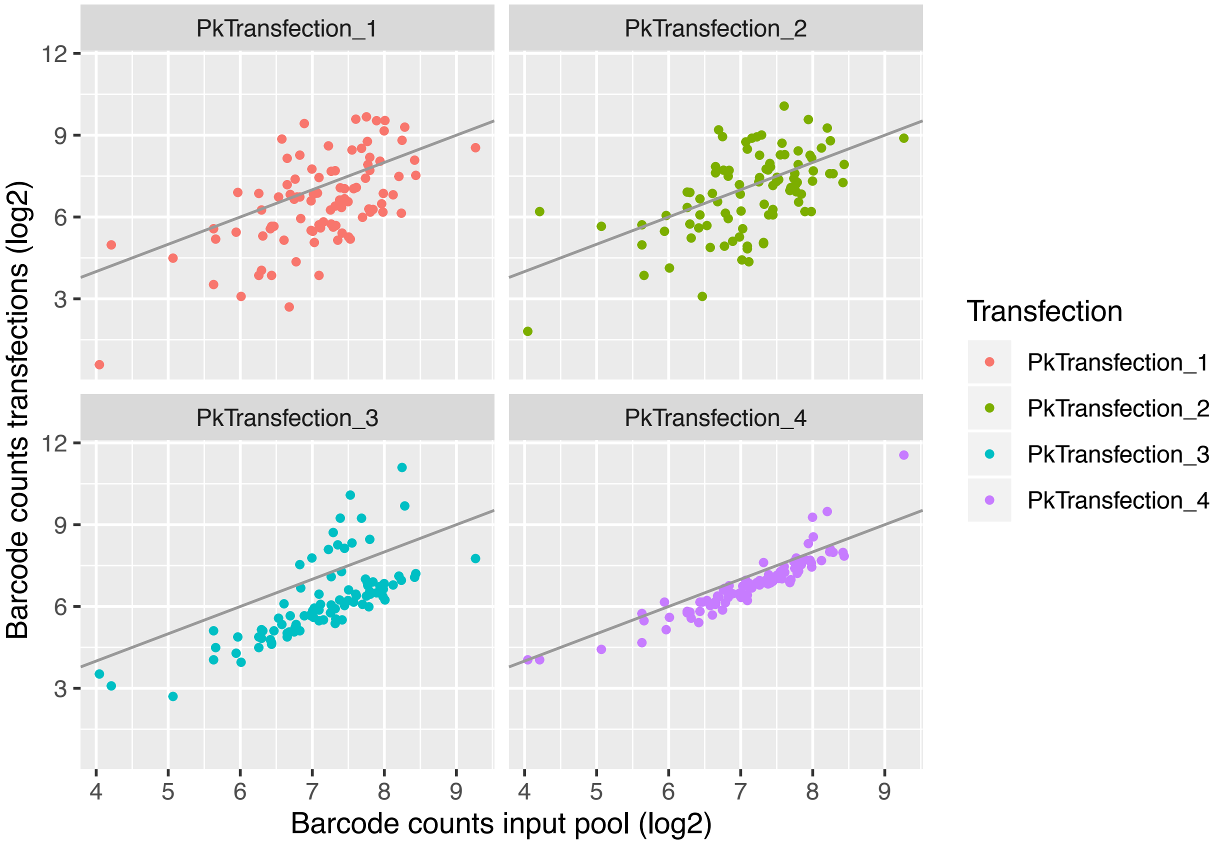


```
ggplot(both,aes(x=diff,color=Transfection))+geom_density() +labs(x="Difference (in log-base-2)",y="Dens
```

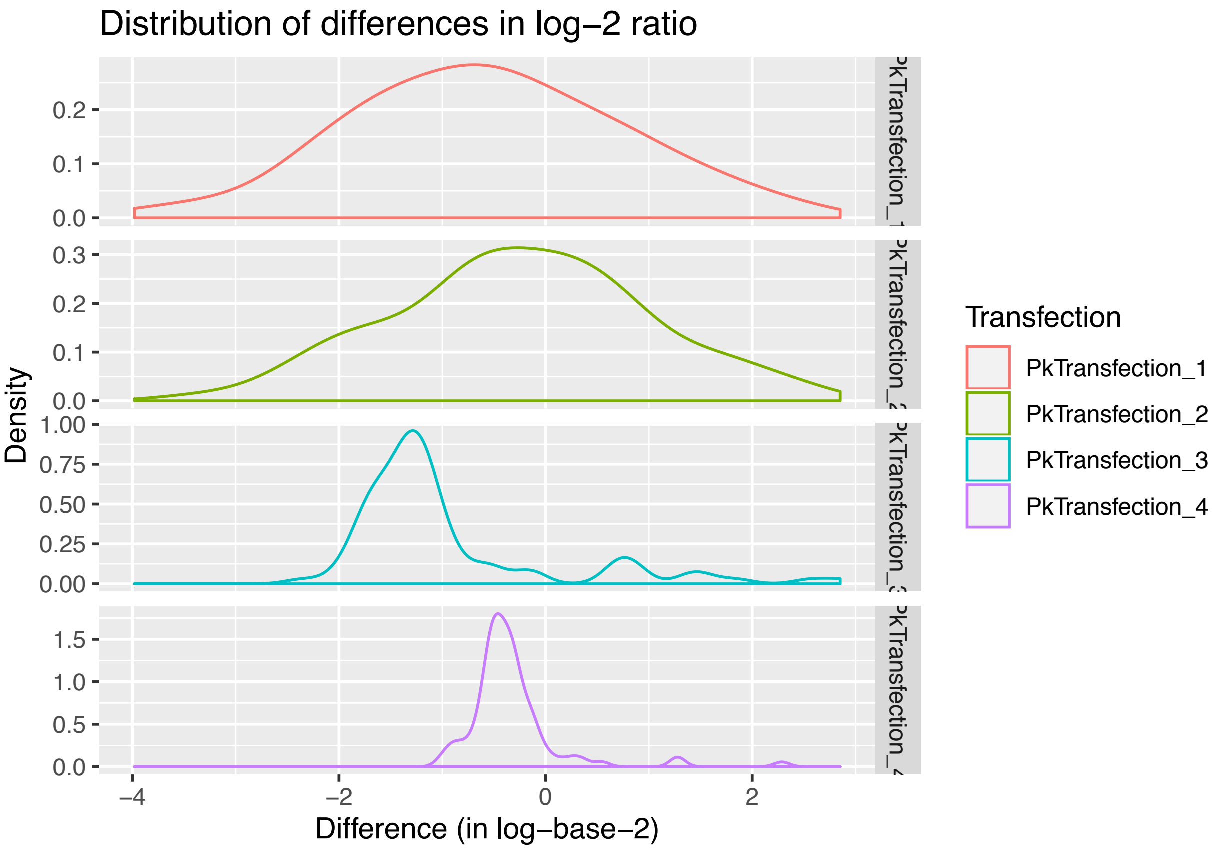

We now have an estimate of the number of unique barcodes in each transfection.

We can now ask another question. Given the proportion of various barcodes in the input, if we simulate a certain number of barcodes being taken up by parasites, then how many molecules would we expect to recover?

```
input2<- narrow %>% filter(type=="Pk_Input") %>% mutate(prop=val/sum(val))
simulate <- function(numberTakenUp){

answer=median(replicate(100,length(unique(sample(x = 1:nrow(input), numberTakenUp, replace = T, prob = 
return(answer)
}
numberTakenUp<-1:4000
numberOfBarcodesRecovered<-sapply(numberTakenUp,simulate)
df<-data.frame(numberTakenUp=numberTakenUp,numberOfBarcodesRecovered=numberOfBarcodesRecovered)
ggplot(df,aes(x=numberTakenUp,y=numberOfBarcodesRecovered))+geom_point()+labs(x="Number of molecules ta
```

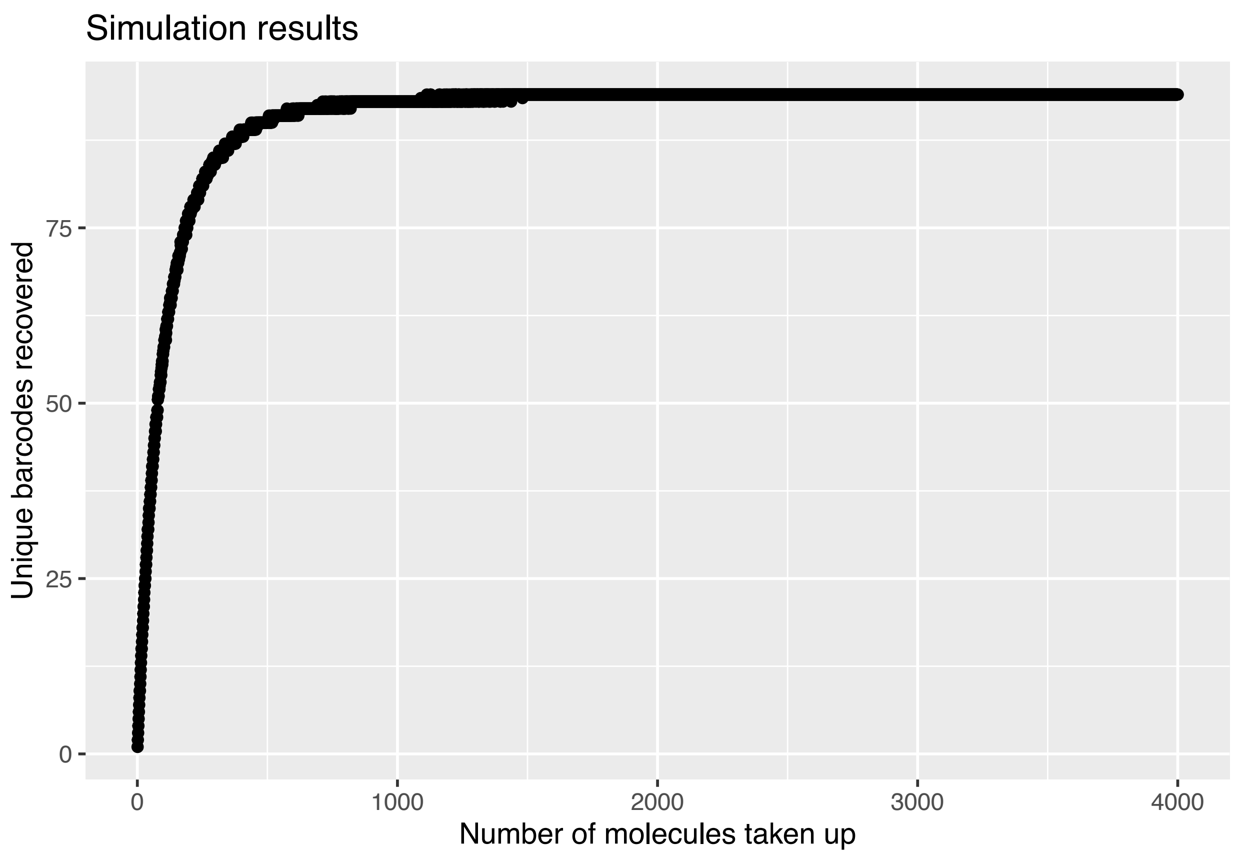


```
df <- df %>% group_by(numberOfBarcodesRecovered) %>% summarise(numberTakenUp=mean(numberTakenUp))
```

We can now use this data to estimate how many molecules of DNA were taken up in each transfection.

```
print(filter(df,numberOfBarcodesRecovered == round(numberOfBarcodesRecovered)), n=100);
```

```
## # A tibble: 93 x 2
##    numberOfBarcodesRecovered numberTakenUp
##                        <dbl>         <dbl>
##  1                         1           1
##  2                         2           2
##  3                         3           3
##  4                         4           4
##  5                         5           5
##  6                         6           6
##  7                         7           7
##  8                         8           8
##  9                         9           9.5
## 10                        10          11
## 11                        11          12
## 12                        12          13
## 13                        13          14
## 14                        14          15
## 15                        15          16.5
## 16                        16          18
## 17                        17          19
## 18                        18          20.5
## 19                        19          22
```

```
## 20                             20           23
## 21                             21           24.5
## 22                             22           26
## 23                             23           27
## 24                             24           28.5
## 25                             25           30.5
## 26                             26           32
## 27                             27           33.5
## 28                             28           35
## 29                             29           36.5
## 30                             30           38
## 31                             31           39
## 32                             32           41
## 33                             33           43
## 34                             34           44
## 35                             35           46
## 36                             36           48.5
## 37                             37           50.5
## 38                             38           52.5
## 39                             39           54.5
## 40                             40           56
## 41                             41           58
## 42                             42           60.5
## 43                             43           62.5
## 44                             44           64.5
## 45                             45           66.5
## 46                             46           69.3
## 47                             47           71
## 48                             48           74.5
## 49                             49           77.5
## 50                             51           81
## 51                             52           84
## 52                             53           88
## 53                             54           91
## 54                             55           93
## 55                             56           96
## 56                             57           98.5
## 57                             58          102
## 58                             59          106.
## 59                             60          111
## 60                             61          113
## 61                             62          116.
## 62                             63          120.
## 63                             64          125
## 64                             65          130.
## 65                             66          134.
## 66                             67          139
## 67                             68          144.
## 68                             69          150.
## 69                             70          155.
## 70                             71          161.
## 71                             72          170
## 72                             73          171.
## 73                             74          181.
```

```
## 74                                 75          187.
## 75                                 76          195
## 76                                 77          201.
## 77                                 78          212.
## 78                                 79          224.
## 79                                 80          235
## 80                                 81          245.
## 81                                 82          258.
## 82                                 83          272.
## 83                                 84          289
## 84                                 85          311.
## 85                                 86          333
## 86                                 87          359.
## 87                                 88          388.
## 88                                 89          429.
## 89                                 90          483.
## 90                                 91          560.
## 91                                 92          697.
## 92                                 93         1029.
## 93                                 94         2639.
```
